# Impact of Carbon Tax and Subsidy Policies on Original Equipment Manufacturers and Remanufacturing Companies from the Perspective of Carbon Emissions

**DOI:** 10.3390/ijerph19106252

**Published:** 2022-05-20

**Authors:** Shuiying Zhao, Yi Xu, Conghu Liu, Fangfang Wei

**Affiliations:** 1 School of Mechanical and Electronic Engineering, Suzhou University, Suzhou 234000, China; zsy_szxy@ahszu.edu.cn (S.Z.); liuconghu@ahszu.edu.cn (C.L.); 2 Center for International Education, Philippine Christian University, Manila 1004, Philippines; 3 Business School, Suzhou University, Suzhou 234000, China; 4 Antai College of Economics & Management, Shanghai Jiao Tong University, Shanghai 200031, China; ffwei@sspu.edu.cn; 5 School of Economics and Management, Shanghai Polytechnic University, Shanghai 201209, China

**Keywords:** carbon emission, original equipment manufacturer (OEM), remanufacturing, game model, sustainable development, global warming, government subsidy

## Abstract

To analyze the impact of government carbon tax and subsidy policies on the manufac turing industry in the context of carbon peaking and carbon neutrality. This paper constructs a game model based on two government policies: a “carbon tax” policy for the original product and a “subsidy” policy for the remanufactured product, taking the original product and the remanufactured product as the objects. The policy game model is used to study the impact of carbon taxes, government subsidies, and carbon emissions on product quality, sales, and corporate profits. The results show that under the carbon tax and government subsidy policies, the price of remanufactured products will decrease, the quality will increase, sales will improve, and remanufacturers’ profits will increase; these outcomes are conducive to the development of remanufacturing enterprises. Meanwhile, the price of original products will increase, quality will decrease, sales will decline, and original equipment manufacturers will have to develop and adopt low-carbon technologies to achieve sustainable development. This paper provides decision support for the formulation of government carbon emission policy, and theories and methods for the sustainable development of the manufacturing industry.

## 1. Introduction

The greenhouse effect has led to natural environmental problems such as global warming, melting glaciers, and sea level rises. High carbon dioxide gas emissions are the main cause of global warming. Therefore, the control of carbon emissions and the development of a low-carbon economy have become universal concerns of international significance. The European Union proposed a political commitment to take the lead in achieving “carbon neutrality” by 2050; in 2020, China promised that its CO_2_ emissions would peak by 2030 and that it would achieve carbon neutrality by 2060 [1]. Subsequently, the United States formulated the “Zero Carbon Emission Action Plan.” One of the effective ways to achieve sustainable economic development and “double carbon” goal is to develop the remanufacturing industry. Remanufacturing can effectively realize the reuse of resources, reduce the total carbon emissions of the manufacturing industry, and realize the circular development of the economy. Studies have shown that remanufactured products consume only 30% of raw materials and 40% of energy, their carbon emissions are only 20%, and their costs can be reduced by 50% (opinions of the National Development and Reform Commission, the Ministry of Science and Technology, and the Ministry of Industry and Information Technology to promote the development of the remanufacturing industry [G] 2010) [2]. Therefore, the remanufacturing industry should be vigorously developed to improve the quality of remanufactured products and market acceptance to realize the inevitable path to achieve carbon peak and carbon neutrality [3,4,5,6].

However, to promote the high-quality development of the remanufacturing industry, it is necessary to solve the problem of low consumer recognition of remanufactured products. Ref. [7] used additive technology to improve the design and manufacturing process. Ref. [8] found that using the seven-step problem-solving method and six sigma tools to improve the pass rate of the paint-and-repair process can significantly increase the pass rate of the product. Refs. [9,10].also proposed an integrated optimization control method for a restructuring system that can effectively improve the quality of remanufactured products by 5.27% and reduce costs by 2.76%.

While improving the research and practice of remanufactured products, some scholars have also studied consumer purchase intentions for remanufactured products. For example, Ref. [11] optimized the implementation of the warranty policy of remanufactured products through discrete event simulation, which improved consumer confidence in purchasing remanufactured products. Ref. [12] study of emerging economies, such as Thailand, found that improving products and prices requires government and industry to coordinate to increase consumer willingness to purchase remanufactured products. Ref. [13] pointed out that discounts have a linear effect on the attractiveness of remanufactured products. Green consumers and consumers who think remanufactured products are green usually find remanufactured products to be significantly more attractive. Ref. [14] Monte Carlo simulation uncovered that the relationship between price and customer preference does not monotonically decrease; notably, it demonstrates more of a bell-shaped or triangular function. Refs. [15,16] applied the theory of planned behavior to data collected from Malaysia and compared actual purchase behavior with purchase intention; they found that consumers will happily buy energy-saving products. Ref. [17] applied the fuzzy Delphi method and single-valued civic set to evaluate consumer motivation to purchase remanufactured products and found that remanufacturers should focus on quality and strive to improve product quality to gain a greater competitive advantage. Ref. [18] used a meta-analysis to conduct a statistical synthesis and analysis of factors related to the purchase intention of remanufactured products and found that consumer purchase intention is greatly affected by attitudes and subjective norms. Ref. [19] combined price, government incentives, and environmental benefits with the adjustment effect of consumer attitudes. They uncovered that consumer attitudes toward remanufactured products are important adjustment factors for predicting consumer shifts to remanufactured products.

Regarding production, remanufactured products are more likely to involve fewer carbon emissions than original products. Ref. [20] pointed out that maximizing the remanufacturing of old parts can greatly reduce CO_2_ emissions and energy consumption. Ref. [21] studied the environmental impact of production and transportation in a hybrid manufacturing-remanufacturing system. Ref. [22] found that the government can control collection rates and product distribution to minimize costs and emissions by providing recycling incentives. Ref. [23] found that lower carbon emission caps or higher carbon trading prices encourage producers to collect and remanufacture second-hand items and curb carbon emissions under the total carbon control and carbon trading policies. Ref. [24] analyzed how emission taxes can be levied to realize the inherent economic, environmental, and social benefits. Ref. [25] observed that the US Environmental Protection Agency had taken major measures to reduce the social costs of carbon, and the proposed carbon tax policy has imposed strict constraints on the carbon emissions generated through supply chain operations. Ref. [26] pointed out that a “circular economy” plays an important role in increasing waste and environmental pollution. The low-carbon emission characteristics of remanufactured products allow original equipment manufacturers (OEMs) to obtain better profits under carbon allowances and trading, but investment in low-carbon production technologies should be expanded. Ref. [27] proposed combining economic and carbon emission targets with carbon tax policies to decide the supply chain’s investment in carbon emission reduction technologies. Ref. [28] proved that the pallet cross-docking policy can reduce carbon emissions. Ref. [29] pointed out the impact of carbon emission policies on the decision to manufacture, remanufacture, and recycle used items and the return rate related to prices; compared with manufacturing, remanufacturing proved an effective strategy for reducing carbon emissions. Ref. [30] proposed linking the product life cycle, plastic remanufacturing, and recycling, and combined mechanical and chemical recycling solutions to reduce CO_2_ emissions. Ref. [31] established a closed-loop supply chain model that includes reworking, waste treatment, carbon emissions, carbon emissions control policies, and green technology investment applications to limit carbon emissions in the production stage. 

Some scholars have also separately studied the comprehensive issues of remanufacturing and carbon emission reduction under carbon tax and government subsidy policies. Ref. [31] constructed a manufacturing/remanufacturing game model under outsourcing remanufacturing from the two policies of the government carbon tax and government subsidy, respectively, and comparatively analyzed the impact of the two policies on outsourcing remanufacturing. Ref. [27] researched the impact of carbon tax policy on tire remanufacturing policy and found that the carbon tax incentive system and carbon tax-export tax rebate system are more conducive to promoting remanufacturing and carbon emission reduction than the carbon tax system alone. Ref. [32] studied the impact of the carbon tax on carbon emission reduction. They found that as the second cycle of remanufacturing carbon emission reduction increases, the total carbon emission reduction of manufacturing/remanufacturing is likely to increase or decrease. Ref. [33] discussed remanufacturing and emission reduction strategies under monopoly and competition scenarios. Ref. [34] used a two-stage game model to study the impact of two government subsidies on the remanufacturing supply chain; they found that obtaining government subsidies brings economic benefits and environmental benefits. Ref. [18] established a game model. They considered the issue of supply chain decision-making under the two subsidy methods of no government subsidies and the level of emission reduction when considering the manufacturer’s independent research and development of emission reduction.

Although scholars at home and abroad have achieved certain results, there are still some shortcomings in the literature:

- To improve the quality of remanufactured products, most studies have focused on improving remanufacturing and remanufacturing optimization technology. Still, these approaches increase the production costs of remanufacturing and thus dampen enthusiasm. Therefore, research into continuously improving the internal drive for high-quality remanufactured products is necessary.
- Related research has shown that consumers are sensitive to quality and low-carbon remanufactured products. However, the relationship between the quality of remanufactured products, carbon emissions, remanufacturing production costs, and consumer willingness to purchase needs to be studied in depth to enable the sustainable development of the remanufacturing industry.
- Most studies aim to improve the recycling rate of old products and optimize production to reduce carbon emissions. Few studies on the relationship between OEMs and remanufacturing companies are based on controlling carbon emissions through government subsidies and carbon tax double-incentive measures. The high-quality development of the remanufacturing industry promotes the research, development, and application of OEM low-carbon technologies.

To address the above challenges, this paper proposes a combination of a carbon tax on the excess carbon emissions of the original product and a “subsidy” policy for remanufactured products. The study constructed a game model between OEMs and remanufacturers from carbon emissions and quality. It is assumed that the government formulates carbon taxes and subsidies based on clear carbon emissions and compares and analyzes the price, quality, and sales volume of original products and remanufactured products and changes in corporate profits under the influence of carbon taxes and subsidies. 

For theoretical value, firstly, we try to study the OEM low carbon technology research and development and remanufacturing enterprises to improve product quality, which will likely provide new ideas for the development of a low-carbon manufacturing industry; second, the game model between OEM and remanufacturing enterprises under different government policies is constructed from the perspective of carbon emission and product quality, which reveals the influence of government intervention on the development of the remanufacturing industry and is expected to further enrich the theory of sustainable development of remanufacturing; thirdly, studying the relationship between the government, OEN, and remanufacturing companies and setting the scope of subsidies is expected to enrich the scope of application of research related to the high-quality development of China’s manufacturing industry.

For practical significance, this study provides a decision-making method for the government to draw up a carbon tax and subsidy amount for the manufacturing industry on the one hand, and provides a reference for a production decision of the remanufacturing industry, as well as decision-making support for the government to promote low-carbon development. On the other hand, through the construction of the game model, it is expected to promote the remanufacturing enterprises to improve their sustainable development ability and provide support for China’s manufacturing industry to achieve the goal of “double carbon”.

To accomplish the above research goals, Section 2 of this paper presents the model and its assumptions; Section 3 details the model construction, solution, and results analysis; Section 4 presents the example analysis and discussion, and Section 5 concludes the paper.

## 2. Model

### 2.1. Problem Description

**Figure 1:**
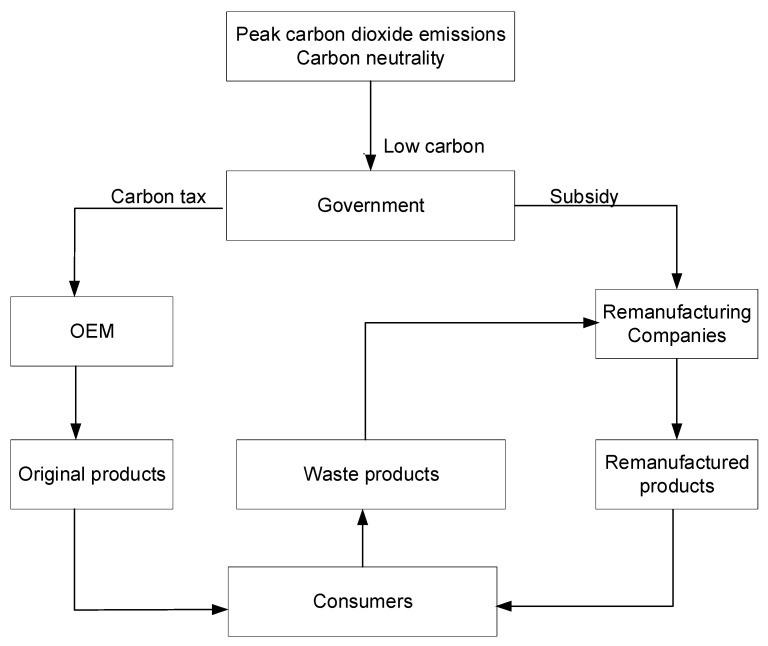
Game model diagram.

Under the carbon peak and carbon neutral target, the government must take measures to reduce carbon emissions from the manufacturing industry. The policy intervenes in the development of the remanufacturing industry through both carbon tax and subsidies, and the government can take two kinds of measures: First, OEMs consume a lot of energy and resources in producing original products, and carbon emissions are high; therefore, the government levies a carbon tax on excess emissions per unit of the original product; second, it involves fewer carbon emissions than producing original products. Therefore, the government provides per-unit subsidies for the carbon emissions saved by remanufactured products to encourage remanufacturing companies to produce more and better remanufactured products to meet market demand and promote the high-quality development of the remanufacturing industry. Consumers purchase original or remanufactured products (considering quality and price) to meet their needs and hand over waste products to remanufacturing companies through reverse logistics. According to the above description, the game diagram of OEM and remanufacturing enterprises under the two government policies can be seen in Figure 1.

### 2.2. Model Notation

#### 2.2.1. Original Product Model Symbols

$c_{0}$: the production cost required to invest in the original product of the production unit;

$p_{0}$: sales price per unit of the original product, $p_{0}>c_{0}$;

$S_{0}$: original product sales

$q_{0}$: the original product quality; the quality cost function is $c_{0}(q_{0})=\frac{k}{2}q_{0}{}^{2}$, *k* is the quality cost coefficient, and the value of *k* is sufficiently large

*t*: the government’s increase in a carbon tax for each original product unit;

$\Delta C_{0}$: carbon emissions per unit of original product exceeding the emission standard

*W_0_*: total revenue from OEM.

#### 2.2.2. Remanufactured Product Model Symbols

$c_{L}$: the production cost that the production unit needs to invest in remanufacturing products, $c_{L}<c_{0}$;

$p_{L}$: unit sales price of remanufactured products, $p_{L}>c_{L}$

$S_{L}$: sales of remanufactured products

$q_{L}$: remanufacturing product quality, the quality cost function is $c_{L}(q_{L})=\frac{k}{2}q_{L}{}^{2}$, *k* is the quality cost coefficient, and the value of *k* is sufficiently large

*v*: the number of government subsidies per remanufactured product unit;

ΔC: relative reduction in carbon emissions per remanufactured product unit;

$W_{L}$: total revenue from remanufacturing companies

### 2.3. Model Assumptions

When consumers buy products, they consider product prices and value product quality. Ref. [35] advise that consumer demand is a function of price and quality. To ensure further research, this study makes the following assumptions:

**Assumption** **1.** *The market demand capacity of the original and remanufactured products is certain. The sales volume of original products is negatively correlated with price and positively correlated with quality.*

**Assumption** **2.** *The sale of original products is also restricted by the price and quality of remanufactured products; the higher the price and the lower the quality of remanufactured products, the greater the sales volume of original products. The lower the price and quality of remanufactured products, the smaller the sales volume of original products.*

**Assumption** **3.** *The same goes for remanufactured product sales.*

Drawing on the literature [1], the original product sales $s_{0}$ and the remanufactured product sales $s_{L}$ are expressed as:

(1) $$S_{0}=\alpha -\beta p_{0}+\gamma p_{L}+\beta q_{0}-\gamma q_{L}$$

(2) $$S_{L}=\alpha -\beta p_{L}+\gamma p_{0}+\beta q_{L}-\gamma q_{0}$$

α represents the market product capacity; β represents the influence coefficient of the product’s own quality and price on sales; and γ represents the substitution parameters of one product to another, assuming β>γ>0.

## 3. Model Formulation and Analysis

### 3.1. Model Formulation

To achieve carbon peak and carbon neutrality, the government needs to adopt effective policies to control carbon emissions from manufacturing. Remanufacturing has been proven to reduce manufacturing costs and carbon emissions. However, carbon taxes have significantly increased the operating costs of companies. To ensure corporate profits and reduce carbon emissions, the government needs to increase carbon taxes on the carbon emissions of original products that exceed the standard and subsidize the reduced carbon emissions of remanufactured products. Due to different product qualities, the investment cost of the enterprise is also different. Among these, the cost includes the production and quality costs. The profits of the OEM and remanufacturing companies *W*_0_ and *W*_L_ are expressed as

(3) $$W_{0}=(p_{0}-c_{0})S_{0}-\frac{k}{2}q_{0}{}^{2}-\Delta C_{0}tS_{0}$$

(4) $$W_{L}=(p_{L}-c_{L})S_{L}-\frac{k}{2}q_{L}{}^{2}+\Delta CvS_{L}$$

Equation (3) $(p_{0}-c_{0})S_{0}-\frac{k}{2}q_{0}{}^{2}$ represents the income obtained by subtracting the cost from the original product sold, and $-\Delta C_{0}tS_{0}$ represents the government’s increased carbon tax on the original products sold for exceeding carbon emissions. Equation (4) $(p_{L}-c_{L})S_{L}-\frac{k}{2}q_{L}{}^{2}$ represents the revenue obtained by subtracting costs from the remanufactured products sold by the remanufacturing company, and $\Delta CvS_{L}$ represents government subsidies for reducing carbon emissions from remanufactured products sold.

### 3.2. Model Solving

**Figure 2:**
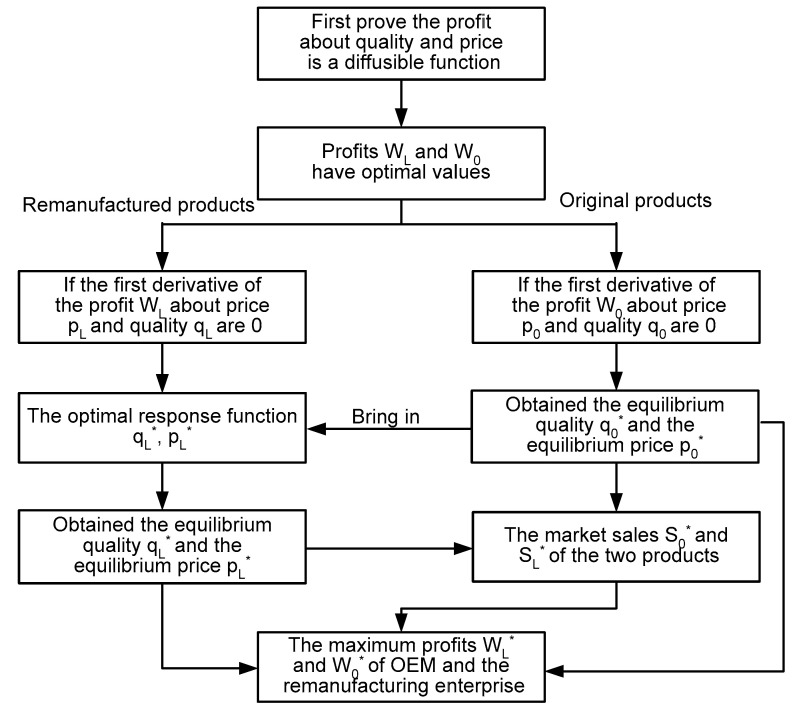
Solving ideas flow diagram.

Although OEM and remanufacturing companies produce the same type of products, there are differences in product quality, carbon emissions, and prices, and the two products have strong substitutes. Since the original product enters the market first after manufacturing, the remanufacturing enterprise will refer to the quality and price of the original product when choosing the product quality and price. Therefore, in the order of the game between OEM and remanufacturing companies, OEM first determines the quality $q_{0}$ and price $p_{0}$ of the original product. At the same time, the remanufacturing enterprise chooses the quality $q_{L}$ and price $p_{L}$ of the remanufactured product according to the decision result of the OEM. The specific solution flow is shown in Figure 2.

Inference 1: Assume (4). The formula for quality and price is a continuous diffusible function.

Prove: Substituting the demand function $S_{L}=\alpha -\beta p_{L}+\gamma p_{0}+\beta q_{L}-\gamma q_{0}$ into Equation (4), we obtain:

(5) $$W_{L}=(p_{L}-c_{L})(\alpha -\beta p_{L}+\gamma p_{0}+\beta q_{L}-\gamma q_{0})-\frac{k}{2}q_{L}{}^{2}+\Delta Cv(\alpha -\beta p_{L}+\gamma p_{0}+\beta q_{L}-\gamma q_{0})$$

In Equation (5), the first derivative and the second derivative of price, $p_{L}$ and quality, $q_{L}$ are $\frac{\partial W_{L}}{\partial p_{L}}=\alpha -2\beta p_{L}+\gamma p_{0}+\beta q_{L}-\gamma q_{0}+\beta c_{L}-\beta \Delta Cv$,$\frac{\partial W_{L}}{\partial q_{L}}=\beta (p_{L}-c_{L})+\beta \Delta Cv-kq_{L}$, $\frac{\partial^{2}W_{L}}{\partial p_{L}{}^{2}}=-2\beta$, $\frac{\partial^{2}W_{L}}{\partial q_{L}{}^{2}}=-k$, $\frac{\partial^{2}W_{L}}{\partial q_{L}\partial p_{L}}=\frac{\partial^{2}W_{L}}{\partial p_{L}\partial q_{L}}=\beta$.

Therefore, its Hessian matrix is $H=\left[\begin{array}{cc} -2\beta & \beta \\ \beta & -k \end{array}\right]$$\left|H_{1}\right|=-2\beta$ < 0, determinant $\left|H\right|=2\beta k-\beta^{2}=\beta (2k-\beta )$ > 0. Therefore, the Hessian matrix is negative definite; the profit function, W_L_, of the remanufacturing enterprise is strictly concave concerning quality $q_{L}$ and price $p_{L}$ and has a unique absolute maximum value; that is, the extreme value of WL represents the maximum profit of the remanufacturing enterprise.

Let $\frac{\partial W_{L}}{\partial p_{L}}$ = 0, and $\frac{\partial W_{L}}{\partial q_{L}}$ = 0, which jointly solve the optimal response function of the product quality and price of the remanufacturing enterprise ($q_{L}^{*}$, $p_{L}^{*}$)

(6) $$q_{L}^{*}=\frac{\alpha -\beta c_{L}+\beta \Delta Cv+\gamma p_{0}-\gamma q_{0}}{2k-\beta }$$

(7) $$p_{L}^{*}=c_{L}-\Delta Cv+\frac{k(\alpha -\beta c_{L}+\beta \Delta Cv+\gamma p_{0}-\gamma q_{0})}{\beta (2k-\beta )}$$

It can be seen from the reaction function that the quality of the remanufactured product $q_{L}$ and the price $p_{L}$ are linearly positively correlated with the original product price $p_{0}$ and linearly negatively correlated with the original product quality. The influence of the original product on the quality and price of the remanufactured product depends on the substitution coefficient. When consumers have a higher acceptance of remanufactured products, increasing the price of original products will promote the quality and price of remanufactured products. Meanwhile, increasing the quality of original products will reduce the quality and price of remanufactured products.

Inference 2: assume k>β Equation (3). The formula for quality $q_{0}$ and price $p_{0}$ is a continuous diffusible function.

The proof process is similar to Inference 1 and thus will not be explained here. Let $\frac{\partial W_{0}}{\partial p_{0}}$ = 0, $\frac{\partial W_{0}}{\partial q_{0}}$ = 0. After substituting reaction functions (6) and (7) ($q_{L}^{*}$, $p_{L}^{*}$), the Nash equilibrium price and quality of the original product can be obtained as

(8) $$p_{0}^{*}=\frac{k\alpha [\beta (2k-\beta )+\gamma (k-\beta )]+\beta (k-\beta )[\beta (2k-\beta )+\gamma^{2}](c_{0}+\Delta C_{0}t)+k^{2}\gamma \beta (c_{L}-\Delta Cv)}{\beta^{2}{\left(2k-\beta \right)}^{2}-\gamma^{2}{\left(k-\beta \right)}^{2}}$$

(9) $$q_{0}^{*}=\frac{\alpha \beta [\beta (2k-\beta )+\gamma (k-\beta )]-\beta [\beta^{2}(2k-\beta )-\gamma^{2}(k-\beta )](c_{0}+\Delta C_{0}t)+k\gamma \beta^{2}(c_{L}-\Delta Cv)}{\beta^{2}{\left(2k-\beta \right)}^{2}-\gamma^{2}{\left(k-\beta \right)}^{2}}$$

By incorporating Equations (8) and (9) into (6) and (7), the equilibrium quality and price of the remanufacturing company can be obtained as

(10) $$q_{L}^{*}=\frac{\alpha \beta [\beta (2k-\beta )+\gamma (k-\beta )]+k\gamma \beta^{2}(c_{0}+\Delta C_{0}t)-\beta [\beta^{2}(2k-\beta )-\gamma^{2}(k-\beta )](c_{L}-\Delta Cv)}{\beta^{2}{\left(2k-\beta \right)}^{2}-\gamma^{2}{\left(k-\beta \right)}^{2}}$$

(11) $$p_{L}^{*}=\frac{k\alpha [\beta (2k-\beta )+\gamma (k-\beta )]+k^{2}\gamma \beta (c_{0}+\Delta C_{0}t)+\beta (k-\beta )[\beta (2k-\beta )+\gamma^{2}](c_{L}-\Delta Cv)}{\beta^{2}{\left(2k-\beta \right)}^{2}-\gamma^{2}{\left(k-\beta \right)}^{2}}$$

According to the equilibrium price and quality of the two products, namely (8)–(11), the sales market of the two products and the maximum profit of the enterprise are calculated as

(12) $$S_{0}^{*}=\frac{k\alpha \beta [\beta (2k-\beta )+\gamma (k-\beta )]-k\beta [\beta^{2}(2k-\beta )-\gamma^{2}(k-\beta )](c_{0}+\Delta C_{0}t)+k^{2}\beta^{2}\gamma (c_{L}-\Delta Cv)}{\beta^{2}{\left(2k-\beta \right)}^{2}-\gamma^{2}{\left(k-\beta \right)}^{2}}$$

(13) $$S_{L}^{*}=\frac{k\alpha \beta [\beta (2k-\beta )+\gamma (k-\beta )]+k^{2}\beta^{2}\gamma (c_{0}+\Delta C_{0}t)-k\beta [\beta^{2}(2k-\beta )-\gamma^{2}(k-\beta )](c_{L}-\Delta Cv)}{\beta^{2}{\left(2k-\beta \right)}^{2}-\gamma^{2}{\left(k-\beta \right)}^{2}}$$

(14) $$W_{0}^{*}=k\beta (k-\frac{\beta }{2})\frac{{\left\{\alpha [\beta (2k-\beta )+\gamma (k-\beta )]-[\beta^{2}(2k-\beta )-\gamma^{2}(k-\beta )](c_{0}+\Delta C_{0}t)+k\beta \gamma (c_{L}-\Delta Cv)\right\}}^{2}}{{\left[\beta^{2}{\left(2k-\beta \right)}^{2}-\gamma^{2}{\left(k-\beta \right)}^{2}\right]}^{2}}$$

(15) $$W_{L}^{*}=k\beta (k-\frac{\beta }{2})\frac{{\left\{\alpha [\beta (2k-\beta )+\gamma (k-\beta )]+k\beta \gamma (c_{0}+\Delta C_{0}t)-[\beta^{2}(2k-\beta )-\gamma^{2}(k-\beta )](c_{L}-\Delta Cv)\right\}}^{2}}{{\left[\beta^{2}{\left(2k-\beta \right)}^{2}-\gamma^{2}{\left(k-\beta \right)}^{2}\right]}^{2}}$$

### 3.3. Model Analysis

Conclusion 1: Government subsidies, carbon tax credits, and product carbon emissions affect the price and quality of the original product.

(1) Relationship with subsidy amount *v*:
(2) Affected by the size of the carbon tax:
(3) Affected by the original product’s excess carbon emissions: $\frac{\partial p_{0}^{*}}{\partial \Delta C_{0}}>0$, $\frac{\partial q_{0}^{*}}{\partial \Delta C_{0}}<0$
(4) Affected by the reduction of carbon emissions, ΔC in remanufactured products:

It can be seen from conclusion 1 that the original product’s price will decrease with an increase in the subsidy amount and will increase with an increase in the carbon tax amount. This is because the government’s increase in subsidies for remanufactured products will reduce the cost of remanufacturing, lower the price of remanufactured products, and promote increased market demand for remanufactured products. On this basis, an increase in the government’s carbon tax is equivalent to an increase in the cost of original products. To ensure profits, OEMs will increase prices and reduce the quality of original products, which will cause the OEM production scale to shrink. The equilibrium price of the original product increases with an increase in its excess carbon emissions, and the quality decreases with an increase in carbon emissions. The higher the carbon emissions of the original products, the more carbon taxes are levied, the higher the price, and the lower the quality under the condition of the same profit. The equilibrium price and quality of the original products decrease with an increase in carbon emissions from remanufactured products. The more carbon emissions are reduced by remanufactured products, and the more government subsidies are given, the lower the prices and the greater the competitive advantage of remanufactured products.

Conclusion 2: The impact of government subsidies, carbon taxes, and product carbon emissions on remanufactured products’ equilibrium price and quality.

(1) Relationship with government subsidy amount, *v*:
(2) Affected by how much the government levies carbon tax, *t*:
(3) Relationship with the original product’s excess carbon emissions $\Delta C_{0}$:$\frac{\partial p_{L}^{*}}{\partial \Delta C_{0}}>0$, $\frac{\partial q_{L}^{*}}{\partial \Delta C_{0}}>0$
(4) Relationship with carbon emission reduction ΔC in remanufactured products:

It can be seen from conclusion 2 that when the government adopts subsidies, the price of remanufactured products decreases as the subsidy increases, and the quality of the remanufactured product increases as the subsidy increases, reducing product costs, lowering prices, and increasing sales. At the same time, companies will also focus on improving product quality to obtain more subsidies. When the government adopts a carbon tax policy, the price and quality of remanufactured products will increase as the carbon tax quota increases. The price and quality of remanufactured products will increase with an increase in the original product’s excess carbon emissions, because the higher the original product’s excess carbon emissions, the higher the related carbon taxes, which will hinder the promotion of the original product in the market and increase the competitiveness of remanufactured products; subsequently, remanufacturing companies can increase prices and quality to enjoy greater profits. The price of remanufactured products will decrease as their carbon emissions decline, and their quality will increase accordingly. In short, increases in government carbon taxes and subsidies and reductions in carbon emissions promote the development of the remanufactured product market toward controlling carbon emissions.

Conclusion 3: Government policies and product carbon emissions affect the sales of original and remanufactured products.

(1) Relationship with subsidy amount *v*:
(2) Relationship with carbon tax:
(3) Affected by excess carbon emissions from the original product: $\frac{\partial S_{0}^{*}}{\partial \Delta C_{0}}<0$, $\frac{\partial S_{L}^{*}}{\partial \Delta C_{0}}>0$
(4) Affected by the reduction of carbon emissions ΔC in remanufactured products:

From conclusion 3, it can be concluded that the sales of original products will decrease with an increase in government subsidies and carbon taxes. In contrast, the sales of remanufactured products will increase with government subsidies and carbon taxes. Remanufactured products are subject to subsidies and carbon taxes, which reduce prices and improve quality, prompting users to choose remanufactured products more often; this may cause the sales of original products to decline. When the production of an original product exceeds standard carbon emissions, the lower the original product sales, the higher the remanufactured product sales. As carbon emissions increase, more carbon taxes are levied on enterprises, and the resulting higher costs are not conducive to original marketing products.

On the contrary, this process is beneficial to remanufactured products; as remanufactured products reduce carbon emissions, sales of original products decrease, while sales of remanufactured products increase. In other words, the more carbon emissions are reduced, the more government subsidies for remanufactured products lower production costs and product prices. As carbon emissions decrease, the sales volume of remanufactured products increases while the sales volume of original products greatly reduces.

Conclusion 4: The maximum profit of OEM and remanufacturing companies is related to the number of government subsidies, increased carbon taxes, and product carbon emissions.

(1) Impact of subsidy amount *v* on the company’s maximum profit:
(2) Impact of carbon tax *t* on the company’s maximum profit:
(3) Impact of the original product’s excess carbon emissions $\Delta C_{0}$ on the company’s maximum profit:
(4) Impact of the reduction in carbon emissions ΔC in remanufactured products on the company’s maximum profit:

It can be seen from conclusion 4 that the larger the government subsidy and carbon tax amount, the smaller the maximum profit obtained by the OEM, and the larger the maximum profit obtained by the remanufacturing enterprise. Government subsidies for remanufactured products will lead to lower prices, higher quality, increased sales, and greater corporate profits. The more carbon taxes are levied on OEMs, the higher the prices and the lower the sales of original products, and the smaller the profits of OEMs. The maximum profit of OEMs will decrease as excess carbon emissions increase, and the maximum profit of remanufacturing companies will increase accordingly. The greater the carbon emissions of the original products, the more carbon taxes are levied, the higher the price of the products, and the lower the sales and profits of the original products; conversely, the greater the carbon emissions of the original production, the higher the quality, price, sales volume, and profit of remanufactured products. Therefore, corporate profits are affected by reducing carbon emissions in remanufactured products: OEMs’ maximum profits will decrease while remanufacturing companies’ maximum profit will increase accordingly. The more remanufacturing companies reduce their carbon emissions, the more they will enjoy subsidies, offer lower prices, and increase sales and profits.

## 4. Case Analysis

In order to verify the correctness of the above model and derived conclusions, this paper uses Python 3.9 to simulate the above propositions to analyze the impact of carbon taxes, subsidies, and carbon emissions on the price, quality, sales volume, and profit of original and remanufactured products.

### 4.1. Government Strategy Influence

According to the research on the relevant remanufacturing product market, the market research on the relevant remanufacturing enterprises, and the relevant variables taken from [31], a remanufactured car engine is 50% cheaper to produce than an original engine. In terms of carbon emissions, the CO_2_ emissions from the original engine manufacturing process are approximately 5*t*, and the CO_2_ emissions from the remanufactured engine manufacturing process are approximately 1*t*—a reduction of nearly 80%, which significantly reduces coal and natural gas usage by 78.5% and 74.4%, respectively. The simulation experiment parameters were selected accordingly. The production costs of the original and remanufactured products were $c_{0}$ = 2 and $c_{L}$ = 1; the quality cost coefficient, *k*, was 10. Suppose the government allows the original product carbon emissions to be 2*t*; the original product will then exceed the standard carbon emission ΔC_0_ = 3*t*. The reduced carbon emissions from the remanufactured products will be ΔC = 4*t.* Also, suppose that the product sales market influence coefficients are α = 100, β = 2, and γ = 1.

**Figure 3:**
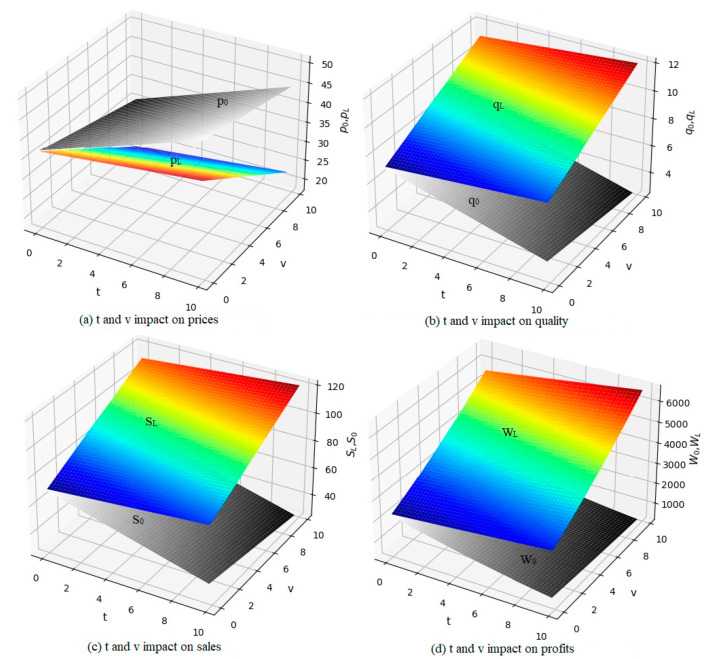
The influence of the government’s t and v on the parameters of the two products.

The experimental results are shown in Figure 3. The results show that both the original product price, *p*_0_, and the remanufactured product price, *p*_L_, are positively correlated with the carbon tax, *t*, and negatively correlated with the subsidy, v. Original product quality, *q*_0_; sales; S_0_; and OEM profit, W_0_, were negatively correlated with the carbon tax and subsidy. Remanufactured product quality, *q*_L_; sales volume, *S_L_*, and remanufacturing company profit, *W_L_*, were positively correlated with carbon tax and subsidy. When the carbon tax and subsidy are larger, the original product’s price will increase and the quality will decrease within a certain range. As a result, market sales and OEM profits were greatly reduced while remanufacturing products had reduced prices and improved quality, which significantly increased the sales and profits of remanufacturing companies.

### 4.2. Product Carbon Emissions Impact

**Figure 4:**
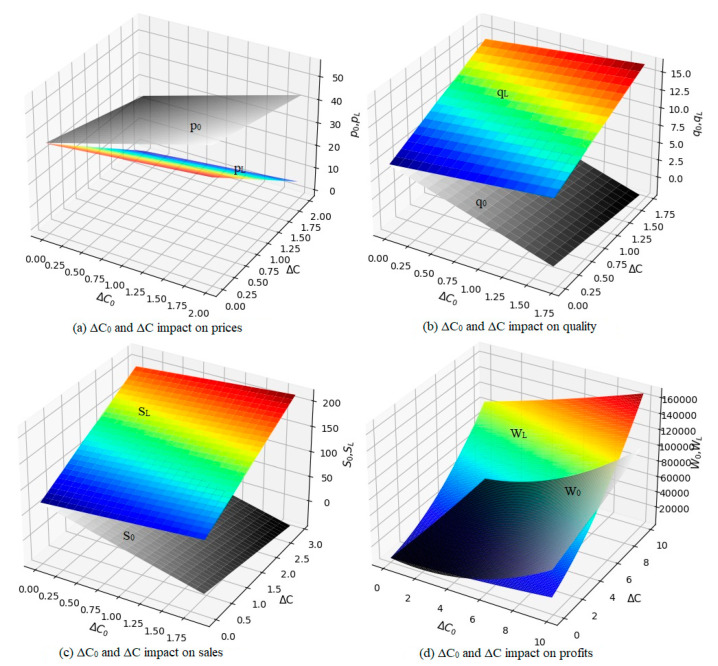
The parameters of the two products are affected by ΔC_0_ and ΔC.

Whether a product is original or remanufactured, carbon emissions vary with quality. For example, when remanufacturing engines are recycled from consumers, the old parts of the engines are classified during disassembly and cleaning into direct replacement parts, direct repair parts, and commissioned repair parts; repaired using repair technology; and finally assembled into a remanufactured engine. During the assembly process, the quality of the parts used and assembly methods are different, and the quality of the remanufactured products produced is also different. Under normal circumstances, the quality of remanufactured products can reach 80% of the original product quality and catch up with or even exceed the original product quality. To achieve the carbon peak and carbon-neutral goals through carbon emissions control in mind, we assume that the government’s increased carbon tax per original product unit exceeded the standard carbon emissions and subsidies for each remanufactured product unit were both 40 yuan/ton. That is, *v* = *t* = 40. The other parameters remained unchanged. The simulations done using Python are shown in Figure 4.

It can be seen from Figure 4 that both the original product price, *p*_0_, and the remanufactured product price, *p_L_*, are positively correlated with the original product’s excess carbon emissions, Δ*C_0_*, and negatively correlated with the remanufactured product’s reduced carbon emissions, Δ*C*. The original product quality, *q_0_*; sales volume, *S_0_*; and OEM revenue, *W_0_*, are negatively correlated with the excess carbon emissions and reduced carbon emissions from remanufactured products. The quality of remanufactured products, *q_L_*; sales, *S_L_*; and revenue, *W_L_*, of remanufacturing companies were positively correlated with their reduced carbon emissions and original product excess carbon emissions. Within a specific value range, when Δ*C_0_* and Δ*C* are larger, the increase in carbon tax for original products and subsidies for remanufactured products will intensify. The price of original products will increase slightly, the quality will decrease, the sales volume will greatly reduce, and OEM profits will reduce. In contrast, the price of remanufactured products is reduced, the quality of remanufactured products greatly improves, the sales volume of remanufactured products significantly increases, and the profits of remanufacturing enterprises significantly increase.

In summary, the simulation results are consistent with the conclusions of the previous theoretical derivation; therefore, the correctness and feasibility of the game model and inference are verified. According to Section 4.1 and Section 4.2, under the influence of government subsidies, carbon taxes, and product carbon emissions, the price of original products is always higher than the price of remanufactured products. Still, the quality of original products may not always be higher than that of remanufactured products. In this situation, the sales and profits of remanufacturing enterprises also exceed OEM sales and profits. Government subsidies can effectively improve the quality of remanufactured products and the expansion of market demand, which is conducive to the development of low-carbon industries. To ensure their market position, OEMs must introduce new technologies, materials, and equipment to reduce their carbon emissions as much as possible, improve product quality, improve product competitiveness, and obtain more profits. In this way, the government can achieve its goal of controlling carbon emissions.

### 4.3. Discussion and Management Implications

Compared with the existing literature [36], this study has several advantages. First, the size of government subsidies and the level of carbon tax are formulated based on the number of carbon emissions, providing a basis for quantifying carbon emissions and determining subsidy and carbon tax rates. As shown in Figure 3, within a certain range, with the increase of government carbon tax rate and government subsidy rate, the sales price of remanufactured products will keep decreasing, the market demand will keep increasing, the quality of products will improve, and the profits of enterprises will increase significantly. Second, providing subsidies to the remanufacturing industry can encourage remanufacturing companies to continuously improve the quality of remanufactured products, promoting the sustainable development of the remanufacturing industry. As shown in Figure 4, under the premise that carbon emissions from the production of different quality products are different, an additional carbon tax is levied on the carbon emissions from the production of the original product that exceeds the limit, and a subsidy of 40RMB/ton is given to the carbon emissions reduced by the production of the remanufactured product. Within a certain range of values, an increase in excess carbon emissions per unit of raw product can lead to a decrease in profits for OEMs, because a higher carbon tax rate is not better. In addition, the more carbon emission reduction per unit of remanufactured products, the higher the amount of government subsidies obtained, and the quality of remanufactured products will continue to improve. As the market demand for remanufactured products continues to increase, the government can also continue to adjust the subsidy tax rate to better promote the high-quality development of the remanufacturing industry. Finally, under subsidies and carbon taxes, OEMs must develop and adopt low-carbon technologies to protect their profits and support carbon emission reduction in the manufacturing industry. As shown in Figure 3 and Figure 4, under the condition that the total market volume remains unchanged, the market demand of the original product will drop due to the squeeze of remanufactured products, the manufacturer of the original product will transfer part of the impact of carbon tax to consumers by increasing the sales price of the product, and the OEM must reduce the carbon emission of the product production in order to reduce the tax burden to achieve sustainable development. The carbon emission of the production of remanufactured products is generally 20% of that of the original product, therefore, vigorously developing the remanufacturing industry can not only reduce the carbon emission of the manufacturing industry, but also provide decision support for the government to achieve the double carbon goal [37,38,39].

This study used a combination of government subsidies and carbon taxes to improve the quality of remanufactured products, develop low-carbon technologies, and encourage resource recycling. The following insights were obtained from the research:

(1) Implications for the government: Choosing appropriate carbon taxes and subsidies based on product carbon emissions is conducive to reducing carbon emissions in manufacturing; promoting the research, development, and application of low-carbon technologies by OEMs; and encouraging the high-quality development of remanufacturing companies.
(2) Implications for OEMs: Under the dual-carbon goal, companies must pay attention to the harmful consequences of carbon emissions for the social environment. To achieve sustainable development, we must engage in the research and development of low-carbon technologies.
(3) Implications for remanufacturing companies: Remanufacturing companies must grasp the “low-carbon” development direction, vigorously improve their equipment, expand their scales of production, apply low-carbon technology, continuously improve the quality of their products, meet market demand, and realize the viability of their industry’s continuous development.
(4) Implications for consumers: To ensure the sustainable use of human living environments and resources, we must acknowledge the importance of going “low carbon,” eliminate prejudice against remanufactured products, and make due contributions to environmental protection and resource conservation.

Overall, carbon emission reduction in manufacturing is a complex project; building a cooperative and win-win carbon emission model requires joint actions of OEMs, remanufacturing companies, the government, and the market. As the leader, the government is duty-bound. OEMs, remanufacturing companies, and consumers should all take up their social responsibilities and contribute to carbon peaking and neutrality.

## 5. Conclusions

Both government carbon tax and subsidy policies are effective tools for government involvement in manufacturing development. The goal of this paper was to analyze the impact of both government policies on manufacturing development in the context of carbon peaking and carbon neutrality. The main contribution of this paper was to propose a game model that adopts a “carbon tax” policy for the original product’s excess carbon emissions and a “subsidy” policy for remanufactured products. The effects of carbon taxes; government subsidies; and carbon emissions on product quality, prices, and corporate profits have been studied. The model’s correctness and effectiveness were verified by analyzing calculation examples. The main conclusions obtained from the study were: Based on the clarification of carbon emission allowance per unit of product, when the government adopts a carbon tax policy, the production of original manufacturing products has higher carbon emissions, and the original manufacturers will increase the sales price of their products to transfer part of the carbon tax to consumers for their own benefit; when the government adopts a subsidy policy, the original manufacturers will obtain a government subsidy by increasing the price of their products to increase their own benefit. Due to the low carbon emissions of remanufactured products in the production process, the government’s adoption of a carbon tax policy can not only effectively promote the development of a remanufacturing industry, but also help reduce the impact of the manufacturing industry on the environment; and when the government adopts a subsidy policy, it can promote the increase of sales of remanufactured products and maintain the high-quality development of the remanufacturing industry.

The above study was conducted under the assumption that there are sufficient waste products. OEMs only produce original products, and remanufacturing enterprises only produce remanufactured products, which has certain limitations. Future research will consider the impact of government policies on manufacturing development in the context of double carbon through patent protection for remanufactured products, outsourcing of remanufacturing production, and uncertainty of consumers’ preference for remanufacturing.

## Data Availability

All data generated or analyzed during this study are included in this published article.
